# Socioeconomic Disparities and Prevalence of Autism Spectrum Disorders and Intellectual Disability

**DOI:** 10.1371/journal.pone.0141964

**Published:** 2015-11-05

**Authors:** Malika Delobel-Ayoub, Virginie Ehlinger, Dana Klapouszczak, Thierry Maffre, Jean-Philippe Raynaud, Cyrille Delpierre, Catherine Arnaud

**Affiliations:** 1 Registre des Handicaps de l’Enfant en Haute-Garonne, CHU de Toulouse, Toulouse, France; 2 INSERM, U1027, Toulouse, France; 3 Université Paul-Sabatier, Toulouse, France; 4 Service Universitaire de Psychiatrie de l’Enfant et de l’Adolescent, CHU de Toulouse, Hôpital La Grave, Toulouse, France; 5 Centre de Ressources Autisme Midi-Pyrénées, Hôpital La Grave, Toulouse, France; 6 Département d’Epidémiologie Clinique, CHU Purpan, Toulouse, France; St Francis Hospital, UNITED STATES

## Abstract

**Background and Objectives:**

Study of the impact of socioeconomic status on autism spectrum disorders (ASD) and severe intellectual disabilities (ID) has yielded conflicting results. Recent European studies suggested that, unlike reports from the United States, low socioeconomic status is associated with an increased risk of ASD. For intellectual disabilities, the links with socioeconomic status vary according to the severity. We wished to clarify the links between socioeconomic status and the prevalence of ASD (with or without ID) and isolated severe ID.

**Methods:**

500 children with ASD and 245 children with severe ID (IQ <50) aged 8 years, born 1995 to 2004, were recruited from a French population-based registry. Inclusions were based on clinical diagnoses reported in medical records according to the International Classification of Diseases, 10^th^ Revision. Socioeconomic status was measured by indicators available at block census level which characterize the population of the child’s area of residence. Measures of deprivation, employment, occupation, education, immigration and family structure were used. Prevalences were compared between groups of census units defined by the tertiles of socioeconomic level in the general population.

**Results:**

Prevalence of ASD with associated ID was higher in areas with the highest level of deprivation and the highest percentage of unemployed adults, persons with no diploma, immigrants and single-parent families. No association was found when using occupational class. Regarding ASD without associated ID, a higher prevalence was found in areas with the highest percentage of immigrants. No association was found for other socioeconomic indicators. The prevalence of isolated severe ID was likely to be higher in the most disadvantaged groups defined by all indicators.

**Conclusion:**

The prevalence of ASD with associated ID and of severe isolated ID is more likely to be higher in areas with the highest level of deprivation.

## Introduction

Recently there has been an increased interest for studies exploring the impact of socioeconomic status (SES) on health status. The social gradient observed for the majority of chronic diseases encourages most countries to consider the reduction of health inequalities as a public health priority. However, results are conflicting regarding the impact of SES on autism spectrum disorders (ASD). The underlying mechanisms are complex and likely related to the health system. Many studies [1–8], mostly American and Australian, have demonstrated an inverse relationship to that usually observed for other health conditions, with a tendency to an increased prevalence of ASD among households with higher SES as measured by parental educational level [1–3, 8] or ecological indicators of household income [1–4, 7]. It has been suggested that these associations primarily reflect a bias in case detection, with an artificially increased prevalence in more favored backgrounds. This hypothesis is supported by some results showing that these links differ according to the source used to measure prevalence and that no relationship is found when cases are identified by educational sources only [1].

On the contrary, the majority of studies [9–11] that found excess risk of ASD in disadvantaged backgrounds have been carried out in European countries. Studies showed an increase in prevalence associated with lower occupational class of parents [11], lower level of maternal education [12] or lower household income [10, 11, 13]. These results were sometimes weakened after adjustment on other risk factors such as perinatal factors [10], or were found only for certain categories such as ASD with low genetic susceptibility [13]. However, the Swedish study of Rai *et al*.[11] reported a clear increase in prevalence in the most deprived backgrounds even after taking into account other associated factors and for all cases of ASD studied i.e. with or without associated intellectual disability (ID).

The joint study of ID and ASD is fully justified by the close links between these two disorders, which involve reciprocal comorbidities and the resulting diagnostic difficulties. The expansion of diagnostic criteria for ASD has also strengthened links with ID. The links between SES and ID prevalence vary according to the severity of ID [5, 9, 14–18]. While moderate ID clearly appears to be more prevalent in children from disadvantaged backgrounds, the results are much less evident for severe ID, with socioeconomic gradients that tend to be less pronounced as the severity of the ID increases [5, 9, 15, 17]. Stromme *et al*. found that children with severe ID were from higher socioeconomic backgrounds than children with less severe forms [17]. In some cases no relationship was found for the more severe forms of ID [16]. Other studies suggested that only isolated ID was associated with SES, unlike ID associated with other serious neurological conditions [15–17].

Given discrepancies and potential for targeting specific groups, the relationship between SES, autism and ID needs to be clarified. We based our study on the data of a population-based registry in France, which has universal and free social health coverage. Area socioeconomic indicators used as proxies of individual SES of families were considered. The objective was to investigate the links between SES and the prevalence of: 1) ASD with or without ID, whatever the severity of ID; 2) severe ID without ASD.

## Methods

### Case Identification

This study included children aged 8 from the childhood disability registry of the administrative area of Haute-Garonne, south-western France. Children born between 1995 and 2004 were selected. The main data source was the local public authority fully responsible for support, guidance, and aid to education for all children living in the surveillance area. Other data sources were child psychiatry departments. Case status was determined by a physician after a comprehensive review of all clinical records available after parental approval as required by national law. For the main administrative data source the informed consent of the parents of all children being registered was sought before consulting the medical record, independently of whether their child was diagnosed with ASD or not. Only a few families (4.6%) refused to allow registry medical staff to consult their child’s medical record, and only some of these records may have involved children with a diagnosis of ASD or severe ID.

Children were included if one of the following diagnoses was reported in medical records. For ASD, the whole ICD-10 F84 category except Rett syndrome was included. Information was collected on intellectual functioning which classified 95% of children with ASD as being with or without an associated intellectual disability (defined as Intellectual Quotient (IQ) <70). For ID without ASD, we considered children with moderate, severe or profound mental retardation according to ICD-10 (corresponding to an IQ level <50) and who were not classified as ASD cases. Diagnosis of intellectual disability was determined either with standardized intelligence tests, mainly Wechsler’s test, or by clinical assessment done by the specialized team responsible for the child and reported in medical records.

### Socioeconomic Indicators

Due to the lack of individual socioeconomic data, socioeconomic indicators of the population of the residence area of each child, available at block census levels, were used to measure the socioeconomic environment of the child’s family. Parental address was used to geolocate each child in one of the 851 census units of the area of surveillance. Geolocation was not possible for 27 children with ASD and 21 with severe ID, yielding a final sample of 500 children with ASD and 245 children with severe ID.

Six indicators available for each census unit were used to study various components of the socioeconomic environment. We first used the French version of the European Index of Deprivation (EDI) [19], a combination of 10 indicators, measuring deprivation. This is a concept proposed by Townsend [20] that “covers the various conditions, independent of income, experienced by people who are poor”. Higher index values indicate higher levels of deprivation in the area. Then in order to refine and attempt to analyze the effect of certain specific conditions, we investigated five particular indicators which although they are included in the EDI, explore specific concepts: the percentages of 1) unemployed adults, 2) workers among the population aged over 15 years, 3) persons aged 15 years or older with no diploma, 4) immigrants (defined as persons of foreign nationality born abroad and living in France), and 5) single-parent families. These indicators were all provided by the French National Institute of Statistics and Economic Studies (INSEE).

### Analysis

To investigate the association between socioeconomic background and ASD or ID, prevalences were compared in three population groups defined by SES levels, using a procedure based on that of Durkin *et al*.[4]. For each SES indicator in turn, three groups of census units based on the tertiles of their distribution in the general population were defined (after weighting by the number of 8-year-old children resident in each census unit), with the first tertile indicating highest SES. Prevalences per 1,000 eight-year-old children residing in each group of census units and their exact 95% confidence intervals (CI) were then calculated. The population denominator was the number of children at risk for the entire period, that is the number of children aged 8 years between 2003 and 2012. The detailed census data at census unit level that best covered our study period were the 2007 data. The denominator used to calculate prevalence at census unit level was estimated as the number of children aged 8 according to the 2007 census data x 10. This estimation was very good because of the negligible increase of the population aged 8 years during this period.

Prevalence Risk ratios (PRR) and *p*-values were derived from negative binomial regression models to take into account overdispersion of data and were computed in order to compare the prevalences according to socioeconomic characteristics. To make the hypothesized dose-response relationship clearer, the same analysis strategy was utilized for the EDI score but the population was split into quintiles as suggested by the authors [19]. In order to take into account a possible effect of change in prevalence across the time period, we computed prevalence for each birth year. Trends over time were tested using a negative binomial regression model. To test the hypothesis that change in prevalence might differ across groups of census units defined by socioeconomic level, we then performed a regression model by adding in the model a term of interaction between census unit group and birth year. All analyses were replicated for 4 groups of cases: 1) all ASD, 2) ASD without ID (IQ >70), 3) ASD with ID (IQ <70), 4) severe ID (IQ <50) without ASD. Statistical software Stata v.11 (StataCorp, College Station, TX, USA) was used for statistical analyses.

### Ethics Statement

This study was approved by the national data protection agency: the “Commission nationale de l'informatique et des libertés” (CNIL, or National Commission for Data Protection and Liberties). The childhood disability registry is approved by this commission for all its usual activities and it received specific authorization for using mailing addresses to locate each child in a census block. Written informed consent was given to the parents of the children prior to their inclusion in the registry. All patient information was anonymized and de-identified prior to analysis.

## Results

**Fig 1:**
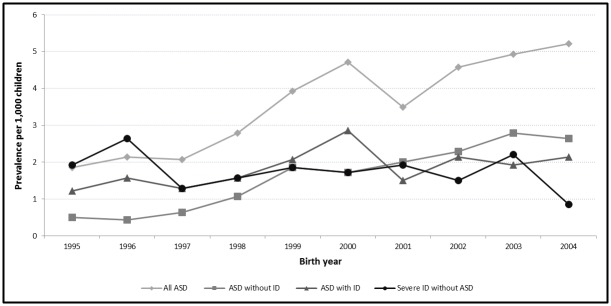
Prevalence of ASD (with and without ID) and severe ID without associated ASD for 1,000 children aged 8 living in the surveillance area and born from 1995 to 2004.

Prevalences of ASD (with or without ID) and severe ID (without ASD) for the entire geographical surveillance area studied are presented in Table 1. The prevalence of ASD was 3.6 per 1,000 (95% CI [3.3–3.9]). Of the 500 children with ASD, 256 (51.2%) also had ID, 223 (44.6%) did not and in 21 (4.2%) cases, information was not sufficient to estimate intellectual functioning. The severe ID without ASD group comprised 245 children, giving a prevalence of 1.8 per 1,000 [1.5–2.0]. Boys were consistently more often affected than girls but this gender difference was reduced when ID was present. Trends of prevalence over time are shown in Fig 1. The prevalence of ASD significantly increased across the period (p<0.001) and this increase was greater for ASD without ID (p<0.001) than for ASD with ID (p = 0.02). For severe ID without associated ASD no trend was observed across this period (p = 0.10). There was no change in the distribution of cases by gender over the period studied.

**Table 1:** Cases Included in the Study and Prevalence of ASD (with and without ID) and Severe ID for 1,000 Eight-Year-Old Children Living in the Surveillance Area between 2003 and 2012.

		Cases included in the study after geolocation in a census unit				
		Denominator ^a^ = 139,930 children				
	N	Boys	Girls	Sex ratio	Prevalence ^b^	95% CI ^c^
All ASD	**500**	404	96	**4.2**	**3.6**	**[3.3–3.9]**
ASD without intellectual disability (IQ >70)	**223**	191	32	**6.0**	**1.6**	**[1.4–1.8]**
ASD with intellectual disability (IQ <70)	**256**	195	61	**3.2**	**1.8**	**[1.6–2.1]**
Severe intellectual disabilities (IQ <50) without ASD	**245**	133	112	**1.2**	**1.8**	**[1.5–2.0]**

Denominator

^a^ = number of 8-year-old children living in the surveillance area between 2003 and 2012 (based on an estimation of the population with 2007 census data carried over to the 10 generations studied).

^b^ *p* = prevalence for 1,000 eight -year-old children living in the surveillance area.

^c^ 95% confidence interval.

**Fig 2:**
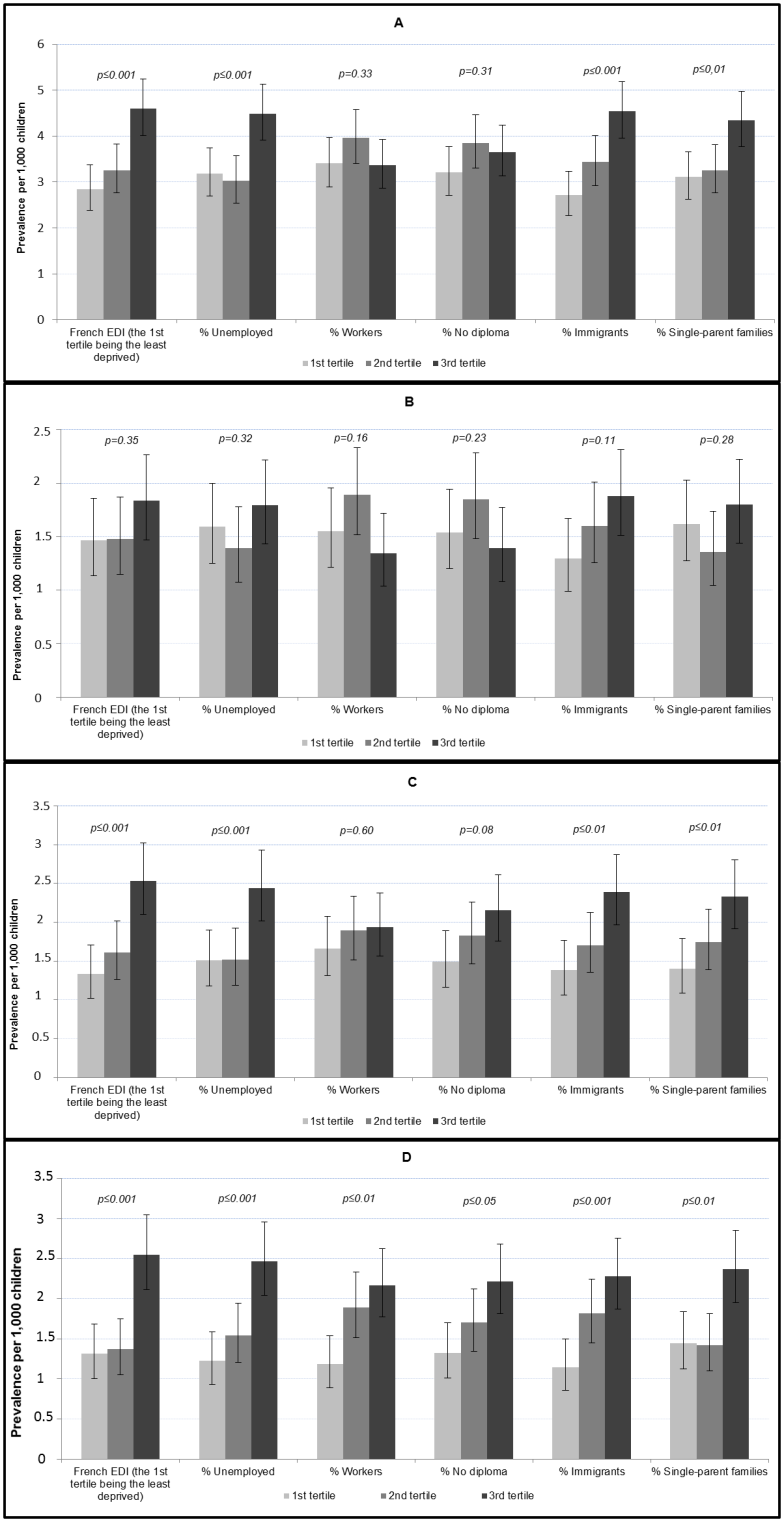
Prevalence (and 95% confidence interval) for 1,000 children aged 8 and born from 1995 to 2004, by six indicators based on census unit data. Census units were divided into tertiles of the 8-year-old population according to the distribution of each indicator in the general population. (A) Prevalence of all ASD (B) Prevalence of ASD without intellectual disability (IQ >70). (C) Prevalence of ASD with Intellectual Disability (IQ <70) (D) Prevalence of Severe Intellectual Disability (IQ <50) without ASD.

Fig 2A–2D shows the prevalence of disorders in the three groups of census units defined by their level of socioeconomic disadvantage. When all ASD were studied together (Fig 2A), prevalence gradually increased with the level of deprivation measured by the EDI (*p* < .001). Prevalence significantly increased with the proportion of immigrants and of single-parent families in the census unit group. Prevalence was also significantly higher in areas with the highest percentage of unemployed adults. No significant association was found when using the proportion of workers or of persons with no diploma. For ASD without ID (Fig 2B), no significant association was found for any of the indicators studied. Results concerning ASD with ID (Fig 2C) were quite similar to those for all ASD. The prevalence of severe ID without associated ASD (Fig 2D) was significantly higher in the census unit groups with the highest EDI score and with the highest proportion of single-parent families. For all other indicators, prevalence gradually increased with the proportions of unemployed adults, workers, persons with no diploma and immigrants in the census unit groups.

Table 2 shows the prevalence risk ratios (PRR) associated with the prevalences presented above, the 1^st^ tertile being considered as the reference. For ASD without ID, there was no obvious trend across the tertiles of SES indicators, except for percentage of immigrants where a significantly increased risk was observed in the 3^rd^ tertile (PRR = 1.44, 95% CI [1.02–2.04]). Regarding ASD with ID, PRR was significantly higher in the most deprived group compared with the reference for the EDI score (PRR = 1.89 [1.39–2.58]), the percentages of unemployed adults (PRR = 1.62 [1.20–2.20]), persons with no diploma (PRR = 1.43 [1.05–1.96]), immigrants (PRR = 1.74 [1.27–2.38]) and single-parent families (PRR = 1.66 [1.22–2.28]). For all these indicators, except for the percentage of unemployed adults, the PRR gradually increased across the groups. No significant association was found with the percentage of workers. For severe ID without associated ASD, PRRs were significantly higher in the most disadvantaged groups compared with the reference for each indicator, with PRRs between 1.62 [1.16–2.27] and 2.03 [1.41–2.93]. A significant gradient across SES groups was observed for percentages of workers and immigrants.

**Table 2:** Prevalence Risk Ratio of ASD and Severe ID by Six Indicators based on Census Unit Data. Census units were divided into tertiles according to the distribution of each indicator, the first tertile being the least deprived and used as a baseline for the computing of risk ratios.

	1st tertile		2nd tertile		3rd tertile	
	(n^a^)	PRR^b^	(n)	PRR [95% CI]	(n)	PRR [95% CI]
All ASD						
French EDI	(132)	1	(150)	1.14 [0.89–1.46]	(218)	1.61 [1.28–2.02]
% Unemployed	(148)	1	(139)	0.94 [0.73–1.20]	(213)	1.42 [1.13–1.78]
% Workers	(158)	1	(182)	1.16 [0.92–1.46]	(160)	0.99 [0.78–1.25]
% No diploma	(148)	1	(179)	1.20 [0.95–1.52]	(173)	1.13 [0.89–1.43]
% Immigrants	(126)	1	(159)	1.27 [0.99–1.62]	(215)	1.68 [1.33–2.12]
% Single-parent families	(144)	1	(151)	1.05 [0.82–1.34]	(205)	1.38 [1.10–1.74]
ASD without Intellectual Disability (IQ >70)						
French EDI	(68)	1	(68)	1.01 [0.71–1.44]	(87)	1.24 [0.89–1.73]
% Unemployed	(74)	1	(64)	0.87 [0.61–1.24]	(85)	1.13 [0.81–1.58]
% Workers	(72)	1	(87)	1.21 [0.87–1.69]	(64)	0.87 [0.61–1.24]
% No diploma	(71)	1	(86)	1.21 [0.87–1.69]	(66)	0.91 [0.64–1.29]
% Immigrants	(60)	1	(74)	1.24 [0.87–1.77]	(89)	1.44 [1.02–2.04]
% Single-parent families	(75)	1	(63)	0.83 [0.58–1.18]	(85)	1.09 [0.78–1.52]
ASD with Intellectual Disability (IQ <70)						
French EDI	(62)	1	(74)	1.20 [0.85–1.69]	(120)	1.89 [1.39–2.58]
% Unemployed	(70)	1	(70)	1.00 [0.72–1.41]	(116)	1.62 [1.20–2.20]
% Workers	(77)	1	(87)	1.14 [0.83–1.57]	(92)	1.16 [0.85–1.59]
% No diploma	(69)	1	(85)	1.22 [0.88–1.69]	(102)	1.43 [1.05–1.96]
% Immigrants	(64)	1	(79)	1.23 [0.88–1.73]	(113)	1.74 [1.27–2.38]
% Single-parent families	(65)	1	(81)	1.26 [0.90–1.76]	(110)	1.66 [1.22–2.28]
Severe Intellectual Disability (IQ <50) without ASD						
French EDI	(61)	1	(63)	1.06 [0.72–1.55]	(121)	1.93 [1.38–2.71]
% Unemployed	(57)	1	(71)	1.25 [0.85–1.83]	(117)	1.98 [1.40–2.81]
% Workers	(55)	1	(87)	1.65 [1.13–2.39]	(103)	1.88 [1.31–2.71]
% No diploma	(61)	1	(79)	1.32 [0.91–1.92]	(105)	1.69 [1.19–2.40]
% Immigrants	(53)	1	(84)	1.59 [1.09–2.31]	(108)	2.03 [1.41–2.91]
% Single-parent families	(67)	1	(66)	1.00 [0.69–1.46]	(112)	1.62 [1.16–2.27]

^a^ n = number of cases in the census unit group defined by tertile of distribution of each indicator in the general population.

^b^ PRR = prevalence risk ratio.

To exclude the possibility that these differences may be partly related to trends in prevalence across the period that may differ according to SES, we tested the interaction with time for the three groups of census units defined by tertile of EDI score. None of the interactions tested were significant (all ASD group p = 0.94; ASD without ID p = 0.67; ASD with ID p = 0.42; severe ID without ASD p = 0.42). We concluded that trends of prevalence over time did not differ in the groups of census units divided according to deprivation level. Moreover, to study to what extent the differences in prevalence between socioeconomic backgrounds may diverge depending on gender, we stratified the analyses. These analyses (S1 and S2 tables) showed that results for ASD followed the same trend towards increased prevalence in the most disadvantaged group for both genders, even if results among girls were less often significant. For severe isolated ID, the differences between the census unit groups were weaker in girls than in boys and were no longer significant.

**Fig 3:**
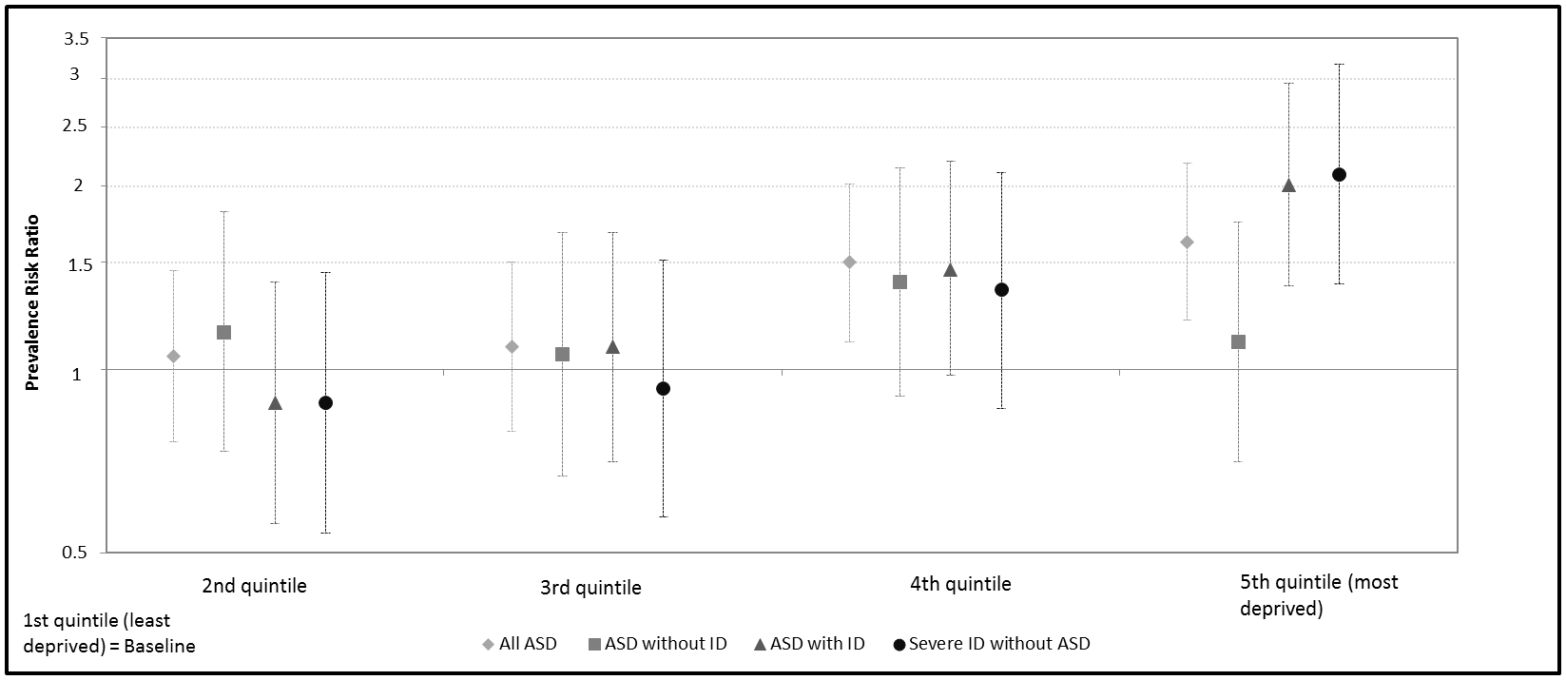
Prevalence risk ratios (and their 95% CI bars) of ASD and severe ID by the index of deprivation based on census block groups of residence divided into population quintiles (the first quintile being the least deprived and used as a baseline).

Fig 3 presents the prevalence risk ratios for each quintile of the EDI score (the first quintile being the least deprived and used as a reference). Results were very similar for the two groups of cases with ID (i.e. children with ASD and associated ID, and children with severe ID without ASD), suggesting that the prevalence significantly increased in the most deprived areas (4^th^ and 5^th^ quintile of census units).

## Discussion

We reported significantly higher prevalences of ASD with ID in areas with the highest level of deprivation and the highest percentage of unemployed adults, persons with no diploma, immigrants and single-parent families. For ASD without ID, a higher prevalence was found only in areas with the highest percentage of immigrants. The prevalence of isolated severe ID tended to increase across the tertiles of socioeconomic level (least deprived to most deprived) captured by all the indicators studied. The two groups of children with ID had similar gradients of risk of elevated prevalence at the highest level of deprivation.

This study was conducted using a population-based database designed to be comprehensive, even if some of the few families that refused to participate may have had a child with an ASD or a severe ID diagnosis. Prevalence rates reported here are similar to those described by the other French childhood disability registry [21, 22], and for ASD they are similar to rates described elsewhere [23–25], even if they appear to be in the lower range of most reports [26–29]. These French prevalences of ASD are presented in more detail in the recent study of van Bakel et al. [22]. The prevalence of ASD increased during the study period, but this trend did not differ according to SES level and so this increase could not explain the main results. This even increase in prevalence for all SES levels also leads us to suppose that changes and improvements in diagnostic practice have benefitted the population as a whole, and tends to disprove the hypothesis of a systematic diagnostic bias associated with SES. However, the increase in prevalence was greater for the group of children without ID for whom the prevalence was not significantly associated with area deprivation. We could thus assume that if the proportion of children without ID continues to increase over time, the result concerning the higher prevalence of ASD as a whole in the most deprived areas might no longer be found. The strength of the recruitment based on clinical records is to provide a prevalence rate of cases that are clinically validated. The age of 8 years at inclusion limited the risk that prevalence may be underestimated because of the children’s young age. However, information may be incomplete for children with less severe forms of ASD. This hypothesis may be supported by the proportion of children with ASD with associated ID, which is higher than that reported elsewhere [11]. The main data source determines all services that can be offered for children with disabilities and is absolutely not limited to financial aid. The cases that may be missing would be children who had not needed any special care, including school aid, before the inclusion age: that is to say, cases of ASD with few clinical signs, and probably some cases of ASD without ID. Thus, the possible under-ascertainment of ASD without ID is unlikely to be linked to the SES of the families and should not bias the results.

We studied the general influence of SES using the EDI index. It has been validated in France and was designed for use in European studies to measure poverty and deprivation [19]. Using the concept of deprivation allows a more complex and much broader study of the notion of disadvantaged background. We completed the measurements of SES using various ecological indicators because there is no consensus about which indicators may be the most relevant. Income, occupation, education and immigration are the most commonly described and are available at the population level. So in our study we did not test the impact of being deprived as an individual but the impact of living in a deprived area.

We found a higher prevalence of ASD in more deprived areas. Associations with low SES have been found for a wide range of indicators as described elsewhere in European or Canadian studies, namely household income [9–11, 13], proportion of single-parent-families [13], or occupational class [11]. Unlike the latter study, we found no association when using the proportion of workers as a proxy of occupational class. However, the professional categories used in France do not have a clear hierarchical relationship and may be difficult to compare with those in other contexts. Stratified analysis by gender led to the same conclusions, although results among the small group of girls obviously had more limited power and were less often significant.

Prevalence of ASD was significantly higher in the most deprived areas only when associated with ID. This association seems to play a key role in the influence of SES on the observed prevalence of these disorders. Taking this interaction into account, our results tended to be intermediate to those previously published. Indeed, Rai *et al*.[11] showed a link between disadvantaged background and increase in prevalence of ASD regardless of associated ID. In contrast, some studies reported an inverse relationship with a higher prevalence of ASD in more favored environments but only for ASD without ID [1, 4, 5]. It is interesting to note the continuum with our results, as we showed an increase in prevalence in disadvantaged areas only for cases of ASD with ID. We may hypothesize that our results also demonstrated a "positive" effect of a better socioeconomic situation on ASD without ID, which would offset the excess of risk observed in disadvantaged backgrounds for other ASD cases. Earlier receipt of support in more advantaged backgrounds could partly explain these results. Very early management could reduce the risk of associated ID at a later age. Previous studies have reported more numerous clinical evaluations and earlier age at diagnosis in advantaged socioeconomic backgrounds [7, 30]. Higher maternal age in more advantaged backgrounds could also play a role in earlier recognition and management of disorders. It has been shown that for the same level of severity of autistic traits, there was a lack of diagnosis of ASD in children of younger or primiparous mothers [31]. In our study, we have no information on age at diagnosis because this item is not currently available in a standardized way in medical records. A trend to a decrease in age at diagnosis has been observed in France in recent decades [32], but no study has examined whether this decrease differed according to socioeconomic situation.

The results for isolated severe ID are consistent with those for ASD and suggested even stronger associations with all indicators studied. Unlike most published results showing a decreased gradient when the severity of ID increases [5, 9, 11–17] we found a significant association between low SES and high prevalence of severe forms of ID. Low maternal educational level [14] or low family income [5, 9, 18] were previously reported to be associated with high prevalence of ID. Family structure, which is more rarely studied, appears to be an important indicator associated with the risk of both severe ID and of ASD with ID. Some situations may worsen household income and may be considered at least in part as a proxy of economic context. In line with our results, an Australian study [5] showed that children of single mothers were at increased risk of mild, moderate or severe ID. When stratified by gender, results showed that associations between low SES and high prevalence of severe ID were stronger for boys than for girls suggesting that the impact of SES factors on severe ID prevalence may differ according to gender.

The association between higher prevalence and a higher proportion of immigrants in the areas studied was observed for all sets of cases including ASD without ID, although this relationship was only of borderline significance. This association suggests complex mechanisms which do not appear to relate only to the SES of immigrant populations. The results in the literature are sometimes contradictory and generally inconclusive because they reflect a mixture of concepts, primarily ethnic origin and migrant status. For ASD, the results are variable according to studies and countries. Some studies in USA have shown that prevalence of ASD was particularly lower among Hispanic children compared to non Hispanic white children [33, 34], and one study [35] which specifically studied the ASD prevalence according to parental place of birth has found that prevalence was particularly lower in US-Hispanic children with 2 foreign-born parents. The role of diagnostic practice, under-recognition of ASD symptoms and socioeconomic disparities in access to services were discussed by the authors. On the contrary, some other studies [36–38] have reported an increased risk of ASD in children of migrant parents. Keen et al.[39] illustrated the role of immigration, rather than ethnic origin, in this increased risk. Some authors have suggested that environmental stressors associated with immigration may play a role [38]. Magnusson et al.[38] showed that the risk is highest when the immigration of the mother took place around birth. This emphasizes the fact that immigrant populations generally appear to be in a vulnerable condition, regardless to some extent of their SES.

In conclusion, the prevalence of ASD with associated ID and of isolated ID is more likely to be higher in areas with the highest level of deprivation. These results show that in a country where social health coverage is universal and free, there are still significant socioeconomic inequalities in children’s health. The findings should encourage further analysis of such environmental factors when studying ASD and/or ID, in order to confirm the results at an individual level. These risks can then be better taken into account when planning preventive measures.

## Supporting Information

S1 Table Prevalence Risk Ratio of ASD and Severe ID by Six Indicators based on Census Unit Data among Girls. (DOCX)

S2 Table Prevalence Risk Ratio of ASD and Severe ID by Six Indicators based on Census Unit Data among Boys. (DOCX)

## Abbreviations

ASD: Autism Spectrum Disorder
CI: Confidence Interval
EDI: European Index of Deprivation
ICD-10: International Classification of Diseases 10^th^ Revision
ID: Intellectual Disability
IQ: Intellectual Quotient
PRR: Prevalence Risk Ratios
SES: SocioEconomic Status
