# Supplementary material for: Socioeconomic Disparities and Prevalence of Autism Spectrum Disorders and Intellectual Disability
Source: PLoS One. 2015 Nov 5;10(11):e0141964. doi: 10.1371/journal.pone.0141964 (PMC4635003; doi:10.1371/journal.pone.0141964)
Supplement: S1 Table — (DOCX) [file pone.0141964.s001.docx]

**Table S1 Prevalence Risk Ratio of ASD and Severe ID by Six Indicators based on Census Unit Data among Girls.** Census units were divided into tertiles according to the distribution of each indicator, the first tertile being the least deprived and used as a baseline for the computing of risk ratios.

|  | 1st tertile | |  | 2nd tertile | |  | 3rd tertile | |  | |
| --- | --- | --- | --- | --- | --- | --- | --- | --- | --- | --- |
|  | (n^a^) | PRR^b^ |  | (n) | PRR [95% CI] |  | (n) | PRR [95% CI] |  | |
| All ASD | | | | | | | | | |  |
| French EDI | (23) | 1 |  | (32) | 1.44 [0.83-2.48] |  | (41) | 1.75 [1.04-2.95] |  |  |
| % Unemployed | (24) | 1 |  | (35) | 1.46 [0.86-2.49] |  | (37) | 1.51 [0.90-2.55] |  |  |
| % Workers | (32) | 1 |  | (38) | 1.22 [0.75-1.98] |  | (26) | 0.80 [0.47-1.35] |  |  |
| % No diploma | (25) | 1 |  | (38) | 1.50 [0.89-2.51] |  | (33) | 1.28 [0.76-2.18] |  |  |
| % Immigrants | (20) | 1 |  | (35) | 1.75 [1.00-3.06] |  | (41) | 1.99 [1.15-3.44] |  |  |
| % Single-parent families | (27) | 1 |  | (33) | 1.25 [0.74-2.11] |  | (36) | 1.32 [0.79-2.20] |  |  |
|  |  |  |  |  |  |  |  |  |  |  |
| ASD without Intellectual Disability (IQ >70) | | | | | | | | | |  |
| French EDI | (10) | 1 |  | (11) | 1.13 [0.47-2.71] |  | (11) | 1.08 [0.45-2.57] |  |  |
| % Unemployed | (10) | 1 |  | (12) | 1.20 [0.51-2.83] |  | (10) | 0.98 [0.40-2.38] |  |  |
| % Workers | (7) | 1 |  | (18) | 2.64 [1.09-6.36] |  | (7) | 0.98 [0.34-2.80] |  |  |
| % No diploma | (6) | 1 |  | (17) | 2.80 [1.10-7.15] |  | (9) | 1.46 [0.52-4.12] |  |  |
| % Immigrants | (7) | 1 |  | (14) | 1.98 [0.79-4.96] |  | (11) | 1.51 [0.58-3.94] |  |  |
| % Single-parent families | (13) | 1 |  | (9) | 0.71 [0.30-1.68] |  | (10) | 0.76 [0.33-1.75] |  |  |
|  |  |  |  |  |  |  |  |  |  |  |
| ASD with Intellectual Disability (IQ <70) | | | | | | | | | |  |
| French EDI | (13) | 1 |  | (20) | 1.58 [0.79-3.18] |  | (28) | 2.12 [1.10-4.10] |  |  |
| % Unemployed | (14) | 1 |  | (22) | 1.59 \|0.81-3.10] |  | (25) | 1.75 [0.91-3.37] |  |  |
| % Workers | (23) | 1 |  | (20) | 0.89 [0.79-1.62] |  | (18) | 0.77 [0.49-1.62] |  |  |
| % No diploma | (17) | 1 |  | (21) | 1.22 [0.65-2.32] |  | (23) | 1.32 [0.71-2.48] |  |  |
| % Immigrants | (13) | 1 |  | (20) | 1.53 [0.76-3.08] |  | (28) | 2.07 [1.08-4.01] |  |  |
| % Single-parent families | (12) | 1 |  | (24) | 2.03 [1.01-4.06] |  | (25) | 2.06 [1.04-4.11] |  |  |
|  |  |  |  |  |  |  |  |  |  |  |
| Severe Intellectual Disability (IQ <50) without ASD | | | | | | | | | |  |
| French EDI | (36) | 1 |  | (30) | 0.86 [0.53-1.40] |  | (46) | 1.26 [0.81-1.96] |  |  |
| % Unemployed | (28) | 1 |  | (36) | 1.29 [0.79-2.14] |  | (48) | 1.68 [1.05-2.70] |  |  |
| % Workers | (30) | 1 |  | (40) | 1.38 [0.85-2.24] |  | (42) | 1.38 [0.86-2.23] |  |  |
| % No diploma | (31) | 1 |  | (39) | 1.25 \|0.77-2.03] |  | (42) | 1.33 [0.83-2.14] |  |  |
| % Immigrants | (29) | 1 |  | (37) | 1.26 \|0.77-2.06] |  | (46) | 1.53 [0.95-2.45] |  |  |
| % Single-parent families | (35) | 1 |  | (34) | 0.99 [0.61-1.60] |  | (43) | 1.21 [0.77-1.91] |  |  |

^a^ n = number of cases in the census unit group defined by tertile of distribution of each indicator in the general population

^b^ PRR = prevalence risk ratio
