# Supplementary material for: Socioeconomic Disparities and Prevalence of Autism Spectrum Disorders and Intellectual Disability
Source: PLoS One. 2015 Nov 5;10(11):e0141964. doi: 10.1371/journal.pone.0141964 (PMC4635003; doi:10.1371/journal.pone.0141964)
Supplement: S2 Table — (DOCX) [file pone.0141964.s002.docx]

**Table S2 Prevalence Risk Ratio of ASD and Severe ID by Six Indicators based on Census Unit Data among Boys.** Census units were divided into tertiles according to the distribution of each indicator, the first tertile being the least deprived and used as a baseline for the computing of risk ratios.

|  | 1st tertile | |  | 2nd tertile | |  | 3rd tertile | |  | |
| --- | --- | --- | --- | --- | --- | --- | --- | --- | --- | --- |
|  | (n^a^) | PRR^b^ |  | (n) | PRR [95% CI] |  | (n) | PRR [95% CI] |  | |
| All ASD | | | | | | | | | |  |
| French EDI | (109) | 1 |  | (118) | 1.08 [0.83-1.41] |  | (177) | 1.59 [1.25-2.04] |  |  |
| % Unemployed | (124) | 1 |  | (104) | 0.85 \|0.65-1.12] |  | (176) | 1.41 [1.11-1.79] |  |  |
| % Workers | (126) | 1 |  | (144) | 1.12 [0.87-1.44] |  | (134) | 1.04 [0.80-1.34] |  |  |
| % No diploma | (123) | 1 |  | (141) | 1.14 [0.89-1.48] |  | (140) | 1.09 [0.85-1.41] |  |  |
| % Immigrants | (106) | 1 |  | (124) | 1.19 [0.91-1.55] |  | (174) | 1.65 [1.28-2.11] |  |  |
| % Single-parent families | (117) | 1 |  | (118) | 1.00 [0.77-1.30] |  | (169) | 1.41 [1.10-1.80] |  |  |
|  |  |  |  |  |  |  |  |  |  |  |
| ASD without Intellectual Disability (IQ >70) | | | | | | | | | |  |
| French EDI | (58) | 1 |  | (57) | 0.97 [0.67-1.42] |  | (76) | 1.27 [0.89-1.81] |  |  |
| % Unemployed | (64) | 1 |  | (52) | 0.82 [0.56-1.20] |  | (75) | 1.16 [0.82-1.64] |  |  |
| % Workers | (65) | 1 |  | (69) | 1.05 [0.74-1.49] |  | (57) | 0.86 [0.60-1.24] |  |  |
| % No diploma | (65) | 1 |  | (69) | 1.06 [0.75-1.52] |  | (57) | 0.86 [0.59-1.24] |  |  |
| % Immigrants | (53) | 1 |  | (60) | 1.14 [0.78-1.67] |  | (78) | 1.46 [1.02-2.09] |  |  |
| % Single-parent families | (62) | 1 |  | (54) | 0.85 [0.58-1.24] |  | (75) | 1.16 [0.82-1.65] |  |  |
|  |  |  |  |  |  |  |  |  |  |  |
| ASD with Intellectual Disability (IQ <70) | | | | | | | | | |  |
| French EDI | (49) | 1 |  | (54) | 1.11 [0.75-1.65] |  | (92) | 1.86 [1.31-2.66] |  |  |
| % Unemployed | (56) | 1 |  | (48) | 0.88 [0.59-1.30] |  | (91) | 1.62 [1.15-2.28] |  |  |
| % Workers | (54) | 1 |  | (67) | 1.21 [0.84-1.76] |  | (74) | 1.33 [0.93-1.91] |  |  |
| % No diploma | (52) | 1 |  | (64) | 1.22 [0.84-1.78] |  | (79) | 1.45 [1.01-2.08] |  |  |
| % Immigrants | (51) | 1 |  | (59) | 1.18 [0.80-1.74] |  | (85) | 1.69 [1.18-2.42] |  |  |
| % Single-parent families | (53) | 1 |  | (57) | 1.09 [0.74-1.60] |  | (85) | 1.59 [1.12-2.27] |  |  |
|  |  |  |  |  |  |  |  |  |  |  |
| Severe Intellectual Disability (IQ <50) without ASD | | | | | | | | | |  |
| French EDI | (25) | 1 |  | (33) | 1.34 [0.76-2.35] |  | (75) | 2.93 [1.79-4.81] |  |  |
| % Unemployed | (29) | 1 |  | (35) | 1.21 [0.70-2.08] |  | (69) | 2.27 [1.40-3.69] |  |  |
| % Workers | (25) | 1 |  | (47) | 1.90 [1.11-3.25] |  | (61) | 2.44 [1.46-4.09] |  |  |
| % No diploma | (30) | 1 |  | (40) | 1.38 [0.82-2.35] |  | (63) | 2.00 [1.23-3.26] |  |  |
| % Immigrants | (24) | 1 |  | (47) | 2.08 [1.21-3.60] |  | (62) | 2.68 [1.59-4.52] |  |  |
| % Single-parent families | (32) | 1 |  | (32) | 0.99 [0.58-1.71] |  | (69) | 2.11 [1.32-3.39] |  |  |

^a^ n = number of cases in the census unit group defined by tertile of distribution of each indicator in the general population

^b^ PRR = prevalence risk ratio
